# Distinct firing responses to synthetic synaptic currents in the adult murine reticular and relay thalamus

**DOI:** 10.1152/jn.00052.2025

**Published:** 2025-03-25

**Authors:** Isaac Y. M. Chang, Jeanne T. Paz

**Affiliations:** 1 Gladstone Institute of Neurological Disease, Gladstone Institutes, San Francisco, California, United States;; 2 Neuroscience Graduate Program, University of California San Francisco, San Francisco, California, United States;; 3 Department of Neurology, University of California San Francisco, San Francisco, California, United States;; 4 Kavli Institute for Fundamental Neuroscience, University of California, San Francisco, California, United States

**Keywords:** circuits, electrophysiology, patch clamp, thalamus

## Abstract

Numerous cortical and subcortical inputs innervate the thalamus and robustly control thalamic activity. These synaptic inputs differ in shape and undergo dynamic changes throughout development and disease conditions. How the shape of postsynaptic currents regulates thalamic neuronal firing has been studied mainly in young rodents with immature neural development and function. Here, we use adult mice with mature intrinsic excitability to address this question in two compartments of the thalamus—the nucleus reticularis thalami (nRT) and thalamocortical (TC) relay nuclei. Using whole cell patch-clamp electrophysiology, we simulated synthetic inhibitory (IPSCs) and synthetic excitatory postsynaptic currents (EPSCs), injected them in nRT and TC neurons, and examined how changes in their shape parameters regulated neuronal firing in different electrical states. We found that in response to synthetic IPSCs, TC neurons initiate low-threshold spikes (LTSs) earlier than nRT neurons, and the amplitude of IPSCs regulates the probability of initiating an LTS while the duration of IPSCs regulates the timing at which the LTS initiates. These results show that in the adult thalamus, LTS is regulated by IPSCs similarly to what has been reported for the immature thalamus. In addition, sharp driver-like EPSCs evoke more firing when nRT and TC neurons are silent; whereas slow modulator-like EPSCs evoke more firing when nRT and TC neurons are active. Critically, we have generated a quantitative map of how features of synaptic currents shape neuronal firing in relationship with activity states.

## NEW & NOTEWORTHY

We provide a systematic overview of how the shape parameters (i.e., amplitude, duration, and charge) of synthetic inhibitory and excitatory synaptic currents regulate neuronal firing in the adult murine thalamus across cell types (nRT vs. TC neurons) and electrical states (active vs. silent).

## INTRODUCTION

Known as the “Gateway to The Cortex” (1), the thalamus is a key brain structure that integrates visual, auditory, sensory, motor, and limbic inputs from subcortical structures and transmits them to the cortex. The thalamus consists of a GABAergic shell (2), termed the nucleus reticularis thalami (nRT), wrapped around glutamatergic nuclei that project to cortical regions, termed thalamocortical (TC) relay nuclei. The nRT and TC neurons receive synaptic inputs from both cortical and subcortical origins. Notably, nRT neurons send powerful GABAergic inhibitory inputs to TC neurons and receive glutamatergic excitatory inputs from TC neurons (3). These reciprocal connections regulate oscillatory activity in the thalamus (4). In addition, nRT neurons receive glutamatergic excitatory inputs from the cortex and amygdala (4), GABAergic input from the external globus pallidus (4), and the substantia nigra pars reticulata (5–7). TC neurons receive excitatory glutamatergic inputs from the neocortex and ascending inputs from their respective modalities [e.g., excitatory retinal input for the visual thalamus (8) and inhibitory basal ganglia input for the motor thalamus (9)].

Kinetic properties of synaptic currents can impact neuronal firing. For glutamatergic synapses, fast (5–10 ms) synaptic currents produced by ionotropic receptors are typically associated with driver roles; slow synaptic currents (hundreds of milliseconds to seconds) are typically associated with modulator roles (10–12). Drivers are inputs that synapse onto proximal dendrites and reliably evoke spikes in thalamic neurons; modulators are inputs that synapse primarily onto distal dendrites and control the probability of cortical transmission (for review, see Refs. 10, 12). Driver inputs such as those mediated by ionotropic AMPA receptors in the nRT have a decay time constant as fast as 0.76 ms (13). On the other hand, modulator inputs activated through mGluR (14) or AChR (15) in the nRT produce slow depolarizations for up to minutes.

The kinetics of synaptic currents can change during development and disease conditions. For example, fast glutamatergic currents at the cortico-nRT synapse, mediated by the GluR4 AMPA receptor, play a key role in the feedforward inhibition in the cortico-nRT-TC circuit (13). The loss of these fast currents at the cortico-nRT synapse leads to neuro-developmental disorders associated with absence epilepsy (13). Understanding how changes in synaptic currents regulate neuronal firing provides crucial insights into rhythmogenesis in the thalamus in both normal and pathological conditions.

Careful studies using dynamic clamp or programmed current clamp protocols showed how distinct synaptic conductances [e.g., GABA (16–21), AMPA (20), NMDA (20)] and intrinsic conductances [e.g., transient Ca^2+^ current (I_T_) and hyperpolarization-activated cation current (I_h_) (22)] affect neuronal firing in the developing thalamus. All these conductances were found to regulate different aspects of the timing and features (e.g., duration) of low-threshold spike (LTS) activation in both the nRT and TC neurons and modulate thalamic network oscillations. However, most studies were conducted in young rodents, not adult mice.

In this study, we investigated how the shape parameters (i.e., amplitude, duration, and charge) of synthetic synaptic currents regulate the activity patterns of nRT and TC neurons in adult mice and how their responses vary depending on the state of neuronal activity.

## MATERIALS AND METHODS

### Animals

All protocols were approved by the Institutional Animal Care and Use Committees at the University of California, San Francisco, and Gladstone Institutes. Precautions were taken to minimize stress and the number of animals used in each set of experiments. Adult male (*n* = 4) and female (*n* = 9) (8–16-wk-old) wild-type C57BL/6J mice (ISMR_JAX: 000664) were used for all experiments.

### Ex Vivo Patch-Clamp Electrophysiology

Mice were deeply anesthetized with 4% isoflurane and perfused transcardially with ice-cold sucrose cutting solution (234 mM sucrose, 26 mM NaHCO_3_, 11 mM glucose, 10 mM MgSO_4_, 2.5 mM KCl, 1.25 mM NaH_2_PO_4_, and 0.5 mM CaCl_2_, equilibrated with 95% O_2_ and 5% CO_2_). Brains were rapidly extracted and dissected in ice-cold sucrose cutting solution. For thalamic recordings, 250-μm-thick horizontal slices were prepared. Slices were incubated at 32°C for 30 min and then at 24–26°C for 1 h in artificial cerebrospinal fluid (ACSF) (126 mM NaCl, 26 mM NaHCO_3_, 10 mM glucose, 2.5 mM KCl, 2 mM CaCl_2_, 1.25 mM NaH_2_PO_4_, and 1 mM MgSO_4_, equilibrated with 95% O_2_ and 5% CO_2_).

Recordings were performed as previously described (4, 13, 23, 24). Briefly, slices were transferred to a chamber perfused at a rate of 1.5–2.0 mL/min with ACSF at 24–26°C. nRT and TC neurons were visually identified by differential contrast optics with an Olympus microscope (×60 objective; numerical aperture, 1.1; working distance 1.5 mm; SKU 1-U2M592). nRT neurons were randomly sampled across the entirety of nRT; TC neurons were sampled across the ventrobasal and ventrolateral nucleus of the thalamus. Electrophysiological data were acquired at 100 kHz and filtered at 3 kHz using a Multiclamp 700B amplifier (Molecular Devices) and the pClamp11 software suite (Molecular Devices). Current clamp recordings were made using glass pipettes with resistance 5–8 MΩ, filled with an internal solution (120 mM potassium gluconate, 11 mM KCl, 11 mM EGTA, 10 mM HEPES,1 mM MgCl_2_, and 1 mM CaCl_2_, pH adjusted to 7.4 with KOH, 290 mosmol/kgH_2_O). E_Cl_^−^ was calculated to be −55.6 mV based on the Nernst equation. Liquid junction potentials were calculated to be −12.5 mV and corrected offline. Recordings were done in the presence of DNQX (30 μM, Sigma-Aldrich, no. D0540), dl-2-Amino-5-phosphonopentanoic acid (APV) (100 μM, Sigma-Aldrich, no. A5282), and picrotoxin (100 μM, Sigma-Aldrich, no. P1675).

### Statistical Analyses

General graphing and statistical analyses were performed using Python and Prism (GraphPad). Sample size is defined by the number of observations (i.e., cells). No statistical method was used to predetermine sample size. Data are presented as mean ± standard error of means. Normal distributions of data were not assumed. Animal subjects and cell recordings were randomized within experimental blocks to yield approximately equal sampling of experimental conditions. Repeated measurements were analyzed using two-way repeated-measures ANOVA in Prism. Statistical significance values and sample sizes are described in the figure legends.

## RESULTS

**Figure 1.**
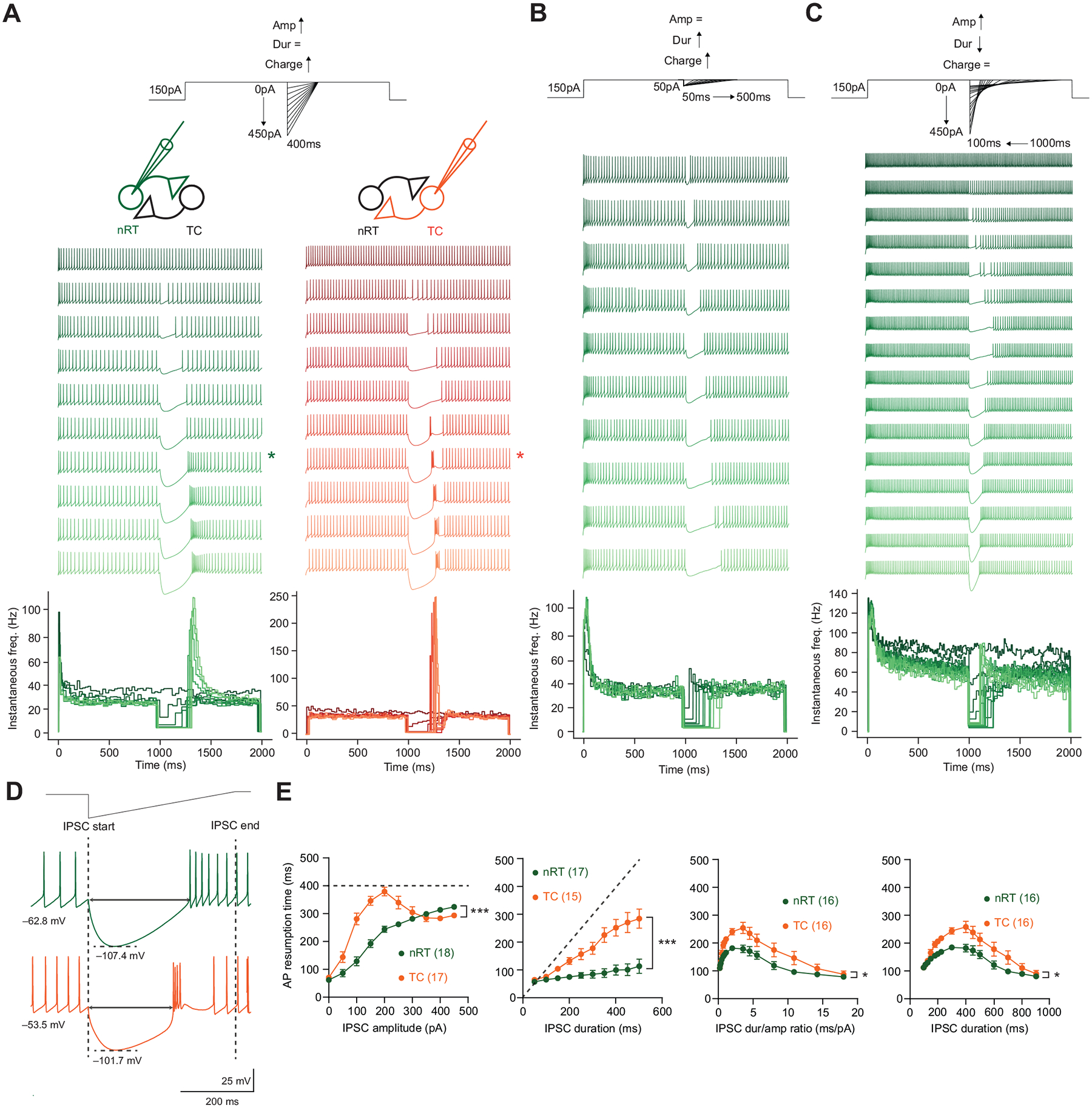
Firing inhibition by synthetic IPSCs during ongoing activity. *A*, *top*: current injection protocol and recording configuration. *Middle*: representative traces of nRT (green) and TC (orange) neurons in response to synthetic IPSCs of increasing amplitude and charge, with the duration held constant at 400 ms. *Traces selected for *D*. *Bottom*: instantaneous firing rate plots. *B*, *top*: current injection protocol. *Middle*: representative traces of nRT neurons in response to synthetic IPSCs of increasing duration and charge, with the amplitude held constant at 50 pA. *Bottom*: instantaneous firing rate plots. *C*, *top*: current injection protocol. *Middle*: representative traces of nRT neurons in response to synthetic IPSCs of increasing amplitude and decreasing duration, with the charge held constant at 22,500 fC. *Bottom*: instantaneous firing rate plots. *D*: zoomed-in traces showing responses to synthetic IPSCs. Dashed lines indicate the start and end of synthetic IPSCs. Arrows indicate the time to LTS. *E*: summary data for results shown in *B* (*left*), *C* (*middle*), and *D* (*right*). Dashed lines indicate the duration of synthetic IPSCs used in the protocols. Two-way RM ANOVA comparing the mean action potential resumption time between nRT (green) and TC (orange) neurons while varying different IPSC shape parameters. IPSC amplitude (*left*) (nRT: *n* = 18 cells, TC: *n* = 17 cells, *P* = 1.30 × 10^−4^); IPSC duration (*middle*) (nRT: *n* = 17 cells, TC: *n* = 15 cells, *P* = 1.58 × 10^−4^); IPSC duration/amplitude ratio (*right*) (nRT: *n* = 16 cells, TC: *n* = 16 cells, *P* = 1.48 × 10^−2^). IPSCs, inhibitory postsynaptic currents; LTS, low-threshold spike; nRT, nucleus reticularis thalami; TC, thalamocortical nuclei. Statistical thresholds used were as follows: *P < 0.05; ***P < 0.001.

A postsynaptic current can be simplified as a triangular waveform characterized by two main parameters: amplitude (height) and duration (width). Charge (area) is a function of amplitude and duration. We describe a sharp brief current (high amplitude, low duration) as a driver-like current; a slow prolonged current (low amplitude, high duration) as a modulator-like current; negative hyperpolarizing currents as inhibitory postsynaptic currents (IPSCs); and positive depolarizing currents as excitatory postsynaptic currents (EPSCs). Triangular current waveforms can be used to simulate synaptic currents with rapid onset and gradual decay. For example, in the nRT, a train of consecutive GABA_A_ currents can be modeled as a triangle with an onset of ~10 ms and a decay of ~500 ms with a peak current ~180 pA (17), and a train of GABA_B_ currents as a triangle with an onset of ~100 ms and a decay of ~1,000 ms with a peak current of ~150 pA (16). The triangular current waveforms used in this study in current clamp have instantaneous onsets, amplitudes, and decay durations in these ranges. These waveforms result in hyperpolarizations (or depolarizations), similar to those evoked by dynamic clamp in the studies cited above. To quantify how changes in shape parameters of IPSCs and EPSCs affect neuronal firing, we simulated synthetic synaptic currents by injecting currents of triangular waveforms during the recording of nRT and TC neurons (Fig. 1A). To isolate the effect on firing by the synthetic synaptic currents without contamination of spontaneous postsynaptic currents, we applied DNQX to block AMPA receptor-mediated currents, APV to block NMDA receptor-mediated currents, and picrotoxin to block GABA_A_ receptor-mediated currents.

We sampled TC neurons across the somatosensory ventrobasal (VB) nucleus and the motor ventrolateral (VL) nucleus and found no significant differences in the firing response characteristics (Fig. 5), passive membrane properties, and action potential characteristics between VB and VL neurons (Table 1). As such, for comparison with nRT neurons, data from VB and VL neurons are combined into a single group (i.e., TC neurons) for all analyses.

### TC Neurons Initiate LTS Earlier than nRT Neurons in Response to IPSCs

To mimic the effect of firing inhibition from an incoming synaptic IPSC during activity, we first injected synthetic IPSCs amid sustained suprathreshold depolarizing current injections. Synthetic IPSCs inhibited firing in nRT neurons, similar to activating afferents from local inhibitory collaterals in the nRT (25) (Fig. 1, A–D). The amplitudes of synthetic IPSCs used range from 0 to 450 pA and are capable of generating hyperpolarizations up to −100 mV, reaching the physiological K^+^-dependent GABA_B_ reversal potential in the nRT (26) and TC (27) neurons. We varied the amplitude and duration of synthetic IPSCs and used the time to the first action potential recovery to measure the duration of the firing inhibition. The duration of firing inhibition scaled with the duration of synthetic IPSCs in both nRT and TC neurons (Fig. 1E, *middle*). However, the duration of firing inhibition scaled with the amplitude of synthetic IPSCs in nRT neurons, but not in TC neurons, which initiated LTS before the full decay of the synthetic IPSC, beyond a certain amplitude threshold during firing (Fig. 1E, *left*). This finding in adult mice aligns with previous studies in young rats showing that, in response to simulated GABA conductances, the onset of LTS is considerably delayed in nRT neurons compared with TC neurons (16, 17). At small and moderate synthetic IPSC amplitudes where LTS are not initiated, TC neurons exhibit more delayed action potential resumption compared with nRT neurons, suggesting that TC neurons are more susceptible to firing inhibition.

What could account for the earlier initiation of LTS in TC neurons, compared with nRT neurons? We quantified the passive membrane properties and action potential characteristics of nRT and TC neurons (Table 1) and found no significant correlations between these properties and the time to LTS (Table 2), suggesting that differences in these properties do not explain why TC neurons initiate LTS earlier than nRT neurons. We speculate that the most likely mechanism underlying this phenomenon is the differential expression of intrinsic membrane currents, i.e., I_h_ and I_T_ currents in nRT and TC neurons, as they have been shown to critically regulate the timing and features of LTS generation (28) (see DISCUSSION).

Next, we varied the amplitude and duration of synthetic IPSCs but kept the total charge of the synthetic IPSCs constant. We asked, given the same total charge, what parameters of synthetic IPSCs most effectively inhibit firing in nRT and TC neurons. We found that a duration/amplitude ratio of 3.31 ± 0.38 and 3.53 ± 0.68 (or when considering duration alone, 450.00 ± 21.89 ms and 462.50 ± 33.39 ms) maximally inhibited firing in both nRT and TC neurons, respectively (Fig. 1E, *right*). The duration/amplitude ratio can be interpreted as a metric for the shape of the synthetic current. An optimal ratio signifies a preferred shape that maximally inhibits firing given the same total charge. The optimal ratios were not significantly different between nRT and TC neurons (Mann–Whitney test, *P* = 0.72 for duration/amplitude ratio, *P* = 0.75 for duration alone). Deviations from this optimal ratio may signify inefficiency in charge transfer.

### IPSC Amplitude Controls The Probability of LTS Initiation; IPSC Duration Controls The Timing of LTS Initiation

**Figure 2.**
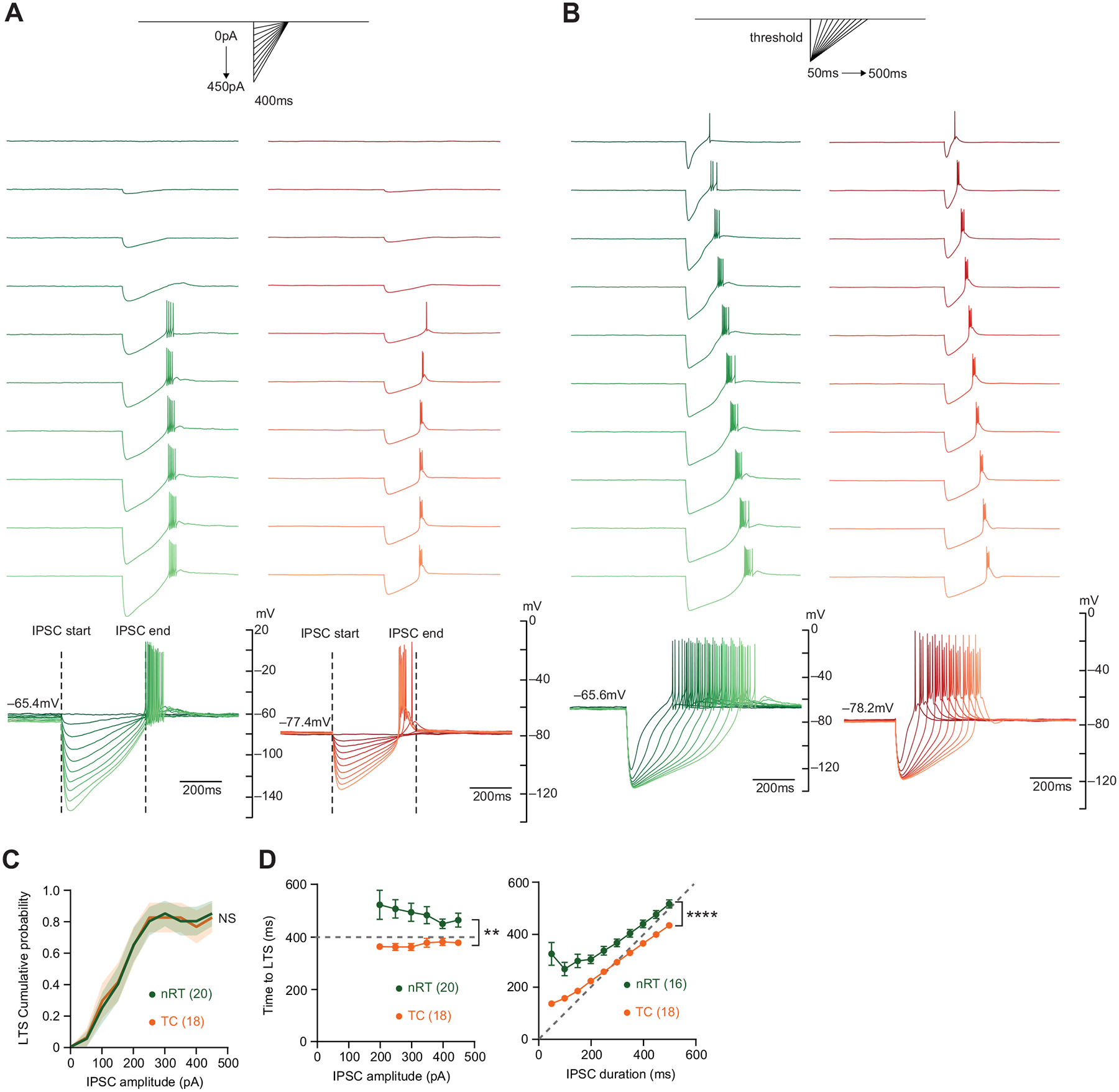
LTS evoked by synthetic IPSCs from rest. *A*, *top*: current injection protocol. *Middle*: representative traces of nRT (green) and TC (orange) neurons in response to synthetic IPSCs of increasing amplitude and charge, with the duration held constant at 400 ms. *Bottom*: traces overlaid. Dashed lines indicate the start and end of synthetic IPSCs. *B*, *top*: current injection protocol. *Middle*: representative traces of nRT (green) and TC (orange) neurons in response to synthetic IPSCs of increasing duration and charge, with the amplitude set at the threshold current, which is defined as the current that evokes LTS at all steps. *Bottom*: traces overlaid. *C*: cumulative probability of evoking LTS with increasing synthetic IPSC amplitude. Two-way RM ANOVA comparing the two cumulative probability curves between nRT (green) and TC (orange) neurons (nRT: *n* = 20 cells, TC: *n* = 18 cells, *P* = 6.57 × 10^−1^). *D*: summary data for results shown in *A* and *B*. Dashed lines indicate the duration of synthetic IPSCs used in the protocols. Two-way RM ANOVA comparing the mean time to LTS from the start of synthetic IPSC between nRT (green) and TC (orange) neurons while varying different IPSC shape parameters. IPSC amplitude (*left*) (nRT: *n* = 20 cells, TC: *n* = 18 cells, *P* = 4.42 × 10^−3^); IPSC duration (*right*) (nRT: *n* = 16 cells, TC: *n* = 18 cells, *P* = 6.23 ×10^−6^). IPSCs, inhibitory postsynaptic currents; LTS, low-threshold spike; nRT, nucleus reticularis thalami; NS, not significant; TC, thalamocortical nuclei. Statistical thresholds used were as follows: **P < 0.01; ****P < 0.0001.

Next, we sought to characterize how nRT and TC neurons respond to synthetic IPSCs at rest (i.e., when they are silent). nRT and TC neurons were generally quiet in brain slices and no specific manipulation was needed to maintain cells in a state of silence. Similar to the approach described above for active states, we measured the time to LTS in nRT and TC neurons while varying the amplitude and duration of synthetic IPSCs (Fig. 2, A and B). We first varied the synthetic IPSC amplitude. The probability of initiating an LTS increased with increasing synthetic IPSC amplitude in both cell types (Fig. 2C). However, the timing of the LTS remained the same regardless of IPSC amplitude (Fig. 2D). A small percentage of nRT (3/20, 15%) and TC (1/18, 5.56%) neurons did not initiate an LTS even when strongly hyperpolarized by synthetic IPSCs of large amplitudes. The nonresponding cells in the nRT were presumably somatostatin-positive nRT neurons, which exhibit smaller peak I_T_ compared with parvalbumin-positive nRT neurons (4).

Next, we varied the synthetic IPSC duration in the same cells. The onset of LTS was delayed with increasing synthetic IPSC duration in both cell types (Fig. 2D). Consistent with our previous experiment, in both conditions, TC neurons produced LTS before the full decay of synthetic IPSCs, whereas nRT neurons produced LTS after the synthetic IPSC had fully decayed (Fig. 2D). The times to LTS in nRT neurons and TC neurons are 465.37 ± 26.47 ms and 378.75 ± 11.41 ms and are significantly different (Mann–Whitney test, *P* = 1.00 × 10^−3^).

Altogether, our results suggest that the initiation of LTS in response to synthetic IPSCs happens earlier in TC neurons than in nRT neurons, and this is a conserved feature across activity states.

### Sharp Driver-Like EPSCs Evoke More Firing When nRT and TC Neurons are Silent; Slow Modulator-Like EPSCs Evoke More Firing When nRT And TC Neurons are Active

**Figure 3.**
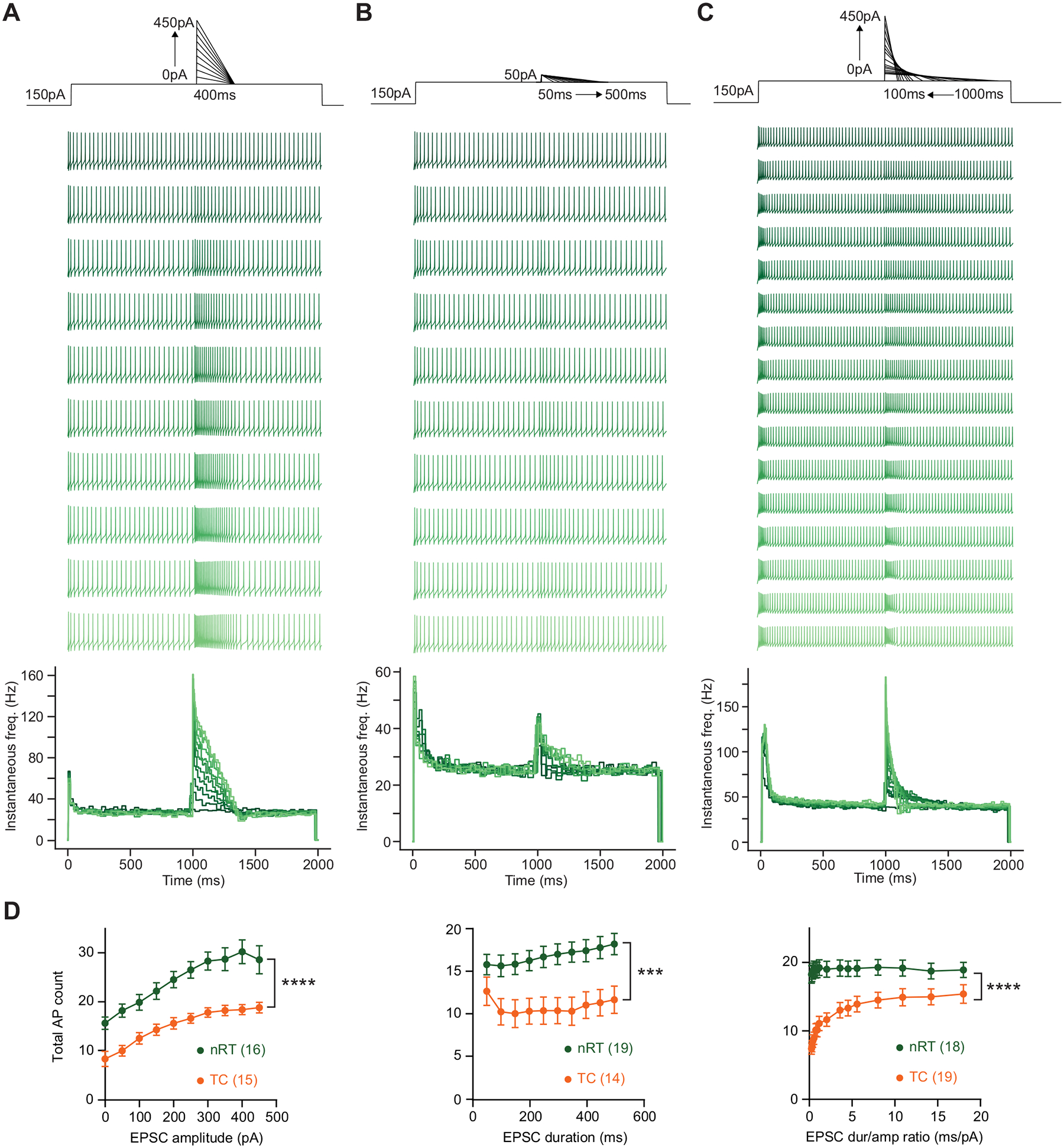
Firing evoked by synthetic EPSCs during ongoing activity. *A*, *top*: current injection protocol. *Middle*: representative traces of nRT neurons in response to synthetic EPSCs of increasing amplitude and charge, with the duration held constant at 400 ms. *Bottom*: instantaneous firing rate plots. *B*, *top*: current injection protocol. *Middle*: representative traces of nRT neurons in response to synthetic EPSCs of increasing duration and charge, with the amplitude held constant at 50 pA. *Bottom*: instantaneous firing rate plots. *C*, *top*: current injection protocol. *Middle*: representative traces of nRT neurons in response to synthetic EPSCs of increasing amplitude and decreasing duration, with the charge held constant at 22,500 fC. *Bottom*: instantaneous firing rate plots. *D*: summary data for results shown in *A* (*left*), *B* (*middle*), and *C* (*right*). Two-way RM ANOVA comparing the mean number of action potentials evoked between nRT (green) and TC (orange) neurons while varying different EPSC shape parameters. The total count of action potentials evoked from the start of the synthetic EPSC to 500 ms after is shown. The start of a synthetic EPSC is 1 s after the start of the depolarizing current injection. EPSC amplitude (*left*) (nRT: *n* = 16 cells, TC: *n* = 15 cells, *P* = 2.75 × 10^−5^); EPSC duration (*middle*) (nRT: *n* = 19 cells, TC: *n* = 14 cells, *P* = 4.34 × 10^−3^); EPSC duration/amplitude ratio (*right*) (nRT: *n* = 18 cells, TC: *n* = 19 cells, *P* = 2.62 × 10^−5^). EPSCs, excitatory postsynaptic currents; LTS, low-threshold spike; nRT, nucleus reticularis thalami; TC, thalamocortical nuclei. Statistical thresholds used were as follows: ***P < 0.001; ****P < 0.0001.

**Figure 4.**
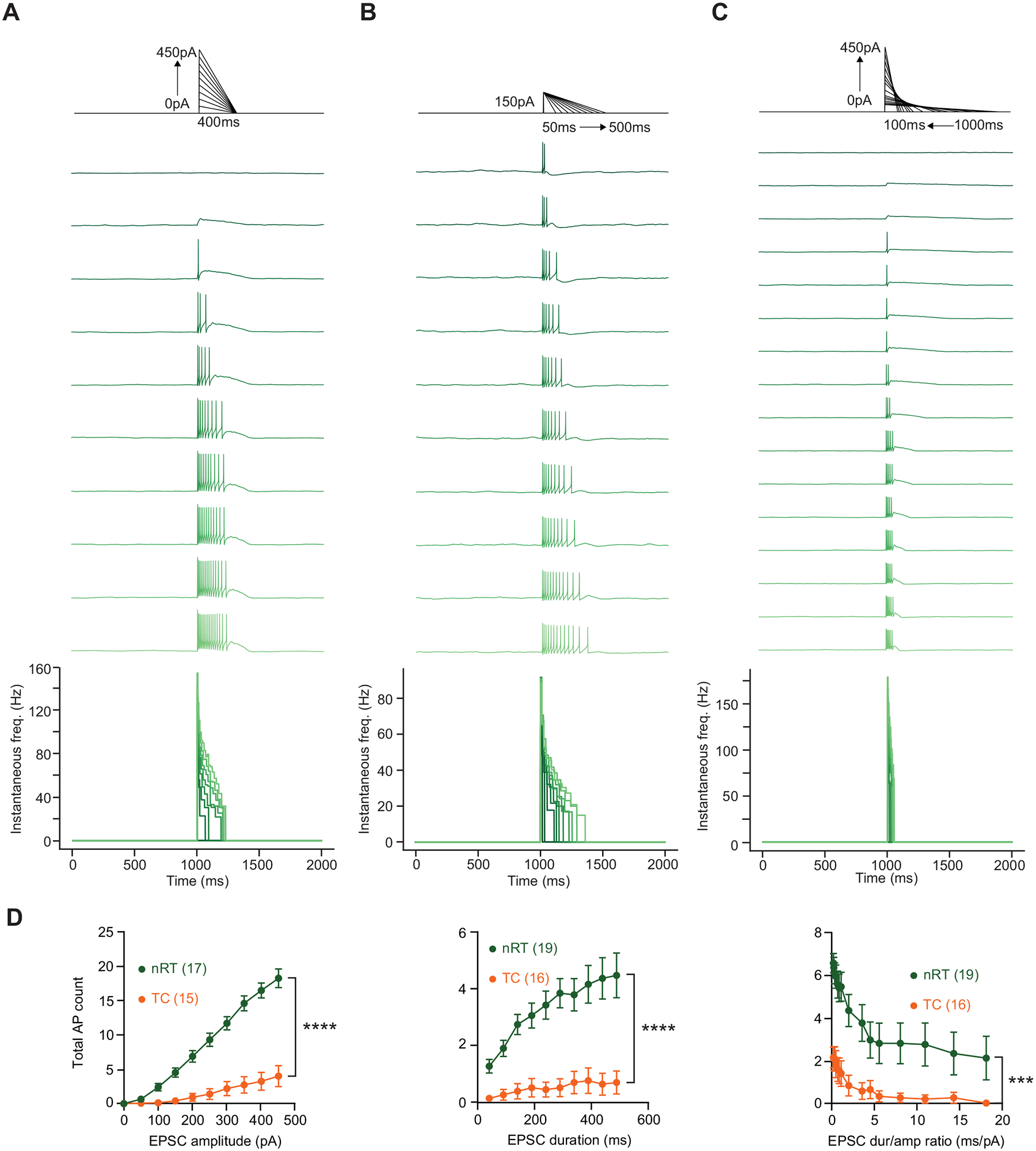
Firing evoked by synthetic EPSCs from rest. *A*: *top*: current injection protocol. *Middle*: representative traces of nRT neurons in response to synthetic EPSCs of increasing amplitude and charge, with the duration held constant at 400 ms. *Bottom*, instantaneous firing rate plots. *B*, *top*: current injection protocol. *Middle*: representative traces of nRT neurons in response to synthetic EPSCs of increasing duration and charge, with the amplitude held constant at 150 pA. *Bottom*: instantaneous firing rate plots. *C*, *top*: current injection protocol. *Middle*: representative traces of nRT neurons in response to synthetic EPSCs of increasing amplitude and decreasing duration, with the charge held constant at 22,500 pA·ms. *Bottom*: instantaneous firing rate plots. *D*: summary data for results shown in *A* (*left*), *B* (*middle*), and *C* (*right*). Two-way RM ANOVA comparing the mean number of action potentials evoked between nRT (green) and TC (orange) neurons while varying different EPSC shape parameters. EPSC amplitude (*left*) (nRT: *n* = 17 cells, TC: *n* = 15 cells, *P* = 7.95 × 10^−8^); EPSC duration (*middle*) (nRT: *n* = 19 cells, TC: *n* = 16 cells, *P* = 4.39 × 10^−5^); EPSC duration/amplitude ratio (*right*) (nRT: *n* = 19 cells, TC: *n* = 16 cells, *P* = 2.75 × 10^−4^). EPSCs, excitatory postsynaptic currents; LTS, low-threshold spike; nRT, nucleus reticularis thalami; TC, thalamocortical nuclei. Statistical thresholds used were as follows: ***P < 0.001; ****P < 0.0001.

**Figure 5.**
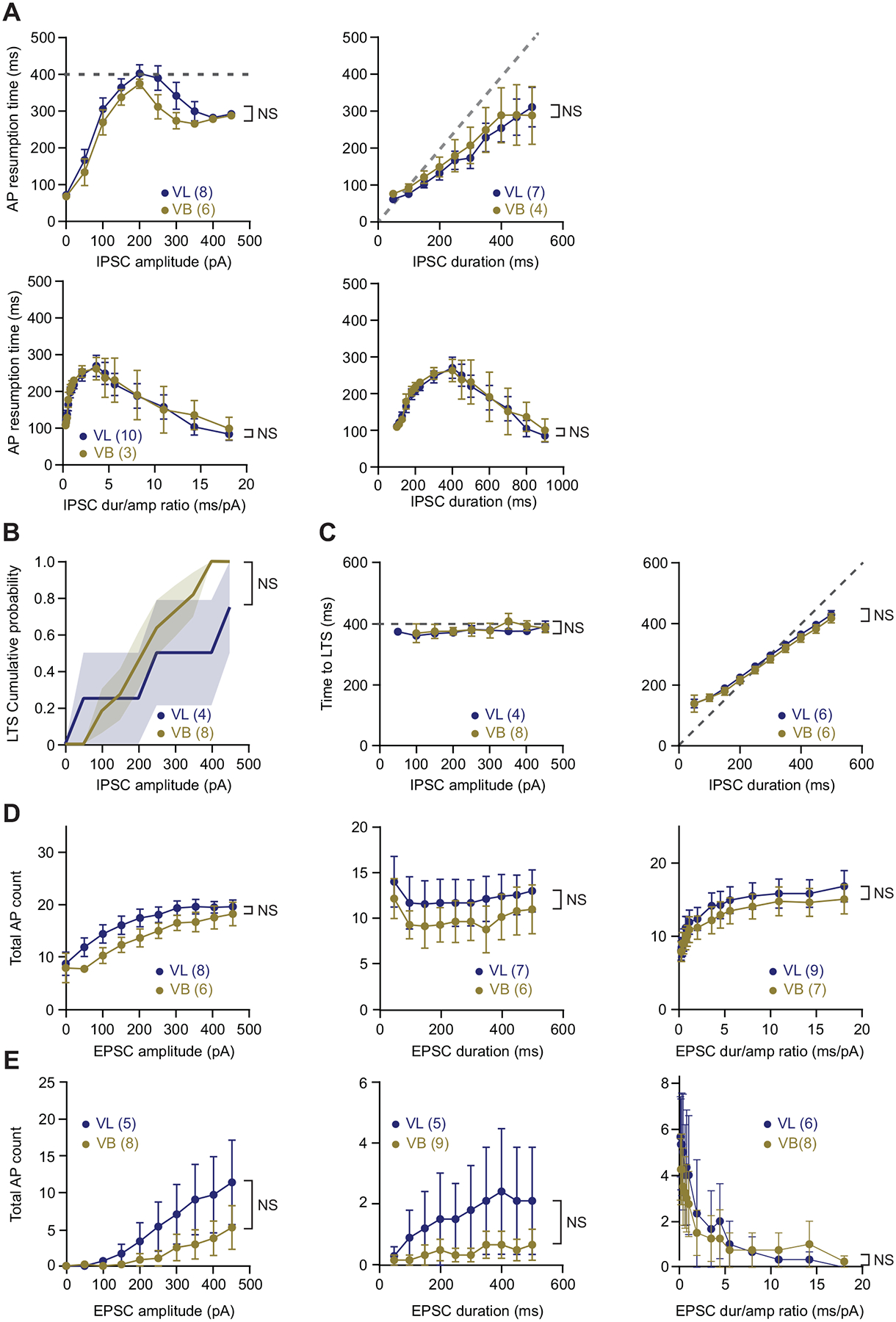
No difference in firing response to synthetic currents between VB and VL neurons. *A*: summary data for results shown in Fig. 1D with TC data split into VB and VL groups. Dashed lines indicate the duration of synthetic IPSCs used in the protocols. Two-way RM ANOVA comparing the mean action potential resumption time between VB (copper) and VL (blue) neurons while varying different IPSC shape parameters. IPSC amplitude (*top left*) (VB: *n* = 6 cells, VL: *n* = 8 cells, *P* = 0.17); IPSC duration (*top right*) (VB: *n* = 4 cells, VL: *n* = 7 cells, *P* = 0.75); IPSC duration/amplitude ratio (*bottom*) (VB: *n* = 3 cells, VL: *n* = 10 cells, *P* = 0.89). *B*: summary data for results shown in Fig. 2C with TC data split into VB and VL groups. Two-way RM ANOVA comparing the two cumulative probability curves between VB (copper) and VL (blue) neurons (VB: *n* = 8 cells, VL: *n* = 4 cells, *P* = 0.41). *C*: summary data for results shown in Fig. 2D with TC data split into VB and VL groups. Dashed lines indicate the duration of synthetic IPSCs used in the protocols. Two-way RM ANOVA comparing the mean time to LTS from the start of synthetic IPSC between VB (copper) and VL (blue) neurons while varying different IPSC shape parameters. IPSC amplitude (*left*) (VB: *n* = 8 cells, VL: *n* = 4 cells, *P* = 0.97); IPSC duration (*right*) (VB: *n* = 6 cells, VL: *n* = 6 cells, *P* = 0.53). *D*: summary data for results shown in Fig. 3D with TC data split into VB and VL groups. Two-way RM ANOVA comparing the mean number of action potentials evoked between VB (copper) and VL (blue) neurons while varying different EPSC shape parameters. EPSC amplitude (*left*) (VB: *n* = 6 cells, VL: *n* = 8 cells, *P* = 0.15); EPSC duration (*middle*) (VB: *n* = 6 cells, VL: *n* = 7 cells, *P* = 0.51); EPSC duration/amplitude ratio (*right*) (VB: *n* = 7 cells, VL: *n* = 9 cells, *P* = 0.65). *E*: summary data for results shown in Fig. 4D with TC data split into VB and VL groups. Two-way RM ANOVA comparing the mean number of action potentials evoked between VB (copper) and VL (blue) neurons while varying different EPSC shape parameters. EPSC amplitude (*left*) (VB: *n* = 8 cells, VL: *n* = 5 cells, *P* =0.23); EPSC duration (*middle*) (VB: *n* = 9 cells, VL: *n* = 5 cells, *P* = 0.30); EPSC duration/amplitude ratio (*right*) (VB: *n* = 8 cells, VL: *n* = 6 cells, *P* = 0.74). EPSCs, excitatory postsynaptic currents; IPSCs, reticularis thalami; NS, not significant; TC, thalamocortical nuclei; EPSCs, inhibitory postsynaptic currents; LTS, low-threshold spike; nRT, nucleus reticularis thalami; NS, not significant; TC, thalamocortical nuclei; VB, ventrobasal nucleus; VL, ventrolateral nucleus.

Finally, we asked how nRT and TC neurons respond to depolarizing synthetic EPSCs during activity and silence. During firing, the number of action potentials evoked increased with injections of synthetic EPSCs of increasing amplitude and duration in nRT neurons, and to a lesser extent, in TC neurons (Fig. 3, A, B, and D). The same phenomenon was observed when nRT and TC responded to synthetic EPSCs at rest (Fig. 4, A, B, and D). The lower excitability of TC neurons compared with nRT neurons could be explained in part by the fact that TC neurons exhibit lower input resistance and more hyperpolarized membrane potential (Table 1) due to the expression of K^+^ channels such as TWIK-related acid-sensitive K^+^ channel 1 (TASK1) and TASK3 (29, 30).

When the total charge of the synthetic EPSC was kept constant, while varying the synthetic EPSC amplitude and duration, we found that, at rest, fast, driver-like EPSCs evoked more firing than slow, modulator-like EPSCs in both nRT and TC neurons (Fig. 3, B and D). Notably, the opposite was true when neurons were active—a slow modulator-like EPSC was more efficient in evoking firing than a fast, driver-like EPSC in both nRT and TC neurons during activity (Fig. 4, B and D). A potential explanation for these results is that when the neuron is silent and resting relatively hyperpolarized, a driver-like EPSC of large amplitude activates T-type Ca^2+^ channels more efficiently than a modulator-like EPSC of small amplitude, leading to more LTS crowned with Na^+^ action potentials.

## DISCUSSION

Our first finding, that TC neurons initiate LTS earlier than nRT neurons in adult mice, corroborates with previous work in young rodents (16, 17), suggesting this is a broadly conserved feature across ages. This observation remains true across activity states, regardless of whether the neuron is active or at rest. Two possible mechanisms could underlie this phenomenon: *1*) activation of I_h_ in TC neurons, which accelerates membrane depolarization to initiate LTS. The possibility of I_h_ is excluded in earlier work, which shows that bath application of Cs^2+^, which blocks I_h_, does not affect the onset of LTS in TC or nRT neurons (17). *2*) A depolarizing shift in the voltage threshold for LTS generation in nRT neurons. Indeed, nRT neurons exhibit distinct T-type Ca^2+^ channel (i.e., Ca_V_3.3), which exhibits slower activation and inactivation kinetics (approximately twice as slow within the LTS generation range of −60 mV to −40 mV), and a more depolarized activation threshold (−50 mV vs. −59 mV) compared with those of TC neurons (i.e., Ca_V_3.1) (31). Therefore, the differential expression of T-type Ca^2+^ channels, but not I_h_, likely explains the difference in the onset of LTS between TC and nRT neurons. An important implication of this difference is that IPSCs generated from intra-nRT and nRT-TC connections could result in different oscillatory frequencies in the nRT and TC nuclei. When an active nRT neuron evokes an IPSC in a connected nRT neuron and TC neuron, the resulting LTS will be considerably delayed in the nRT neuron than in the TC neuron. At a network level, this implies that oscillations within intra-nRT connections would be slower than oscillations within nRT-TC connections due to the longer interval between the IPSCs and LTS generation. Previous in vivo studies in rats showed that the deafferented nRT could generate 7–14 Hz spindle-like oscillations in the slow frequency range via its intra-nRT connections (32). This spindle oscillation in nRT, according to modeling studies, could initiate spindle oscillations in the nRT-TC network (33). However, it remains to be determined empirically whether intra-nRT and nRT-TC networks could operate at distinct frequencies.

We also showed that at rest, IPSC amplitude and duration separately control LTS initiation probability and timing. Contrary to this, a previous study in young rodents has shown that injecting spindle-like trains of IPSCs in TC neurons with increasing amplitude and duration increases LTS initiation probability and decreases time to LTS in response to those trains (19), suggesting that IPSC amplitude and duration interact to control both LTS initiation probability and timing. This difference might be attributed to injections of different IPSC waveforms, as we injected only a single IPSC, while the previous study used trains of IPSCs.

Finally, we found that at rest, sharp driver-like EPSCs evoke more firing when cells are at rest, whereas slow modulator-like EPSCs evoke more firing during activity. Modulatory-like EPSCs can emerge from several sources—application of nor-adrenaline or acetylcholine induces slow depolarizations in TC neurons (34). Pharmacological activation of mGluR in nRT neurons produces a long-lasting excitatory response (14). Another possible source of modulator-like EPSCs in the nRT is depolarizing GABA. Although still controversial, there is evidence to support that GABA is depolarizing in the nRT in certain conditions (35). The nRT shows reduced expression of the Cl^−^ exporter, KCC2, compared with other brain regions in adults, and its physiological ECl^−^ is elevated (35–38). Synaptically released GABA appears to be capable of triggering action potentials in nRT neurons (35). This, coupled with the exceptionally slow decay time constants for GABAergic currents in nRT neurons (τ_fast_ = 38 ms; τ_slow_ = 186 ms) (39), supports the existence of these modulator-like EPSCs in the nRT.

Biological variables such as species, temperature, sex, and age are important determinants of thalamic physiology. For instance, recording at physiological temperature (i.e., 32°C), compared with room temperature, reduces synaptically driven spiking in TC neurons in response to trains of optical stimulation (40). Although our study did not examine the effects of temperature, species differences, or specific thalamic neuron types (e.g., first-order vs. higher-order nuclei), it provides a valuable reference for interpreting synaptic current changes in future studies. Furthermore, neurons in vivo are continuously bombarded by intense synaptic background activity (41). Here, we used a simple paradigm to investigate how a single incoming synthetic synaptic current influences neuronal firing, which does not account for the complex interactions that arise when multiple synaptic currents act in concert to influence spiking behavior. Future studies could expand upon our findings by examining how these factors collectively modulate neuronal activity.

A limitation of the synthetic currents used in our study is that these currents do not account for the reduction of the driving force that would happen with natural synaptic currents as the membrane potential approaches the reversal potential. This would be captured by dynamic clamp. Furthermore, we did not examine how changes in membrane conductance, which would normally be associated with the opening of ion channels, affect firing. Therefore, the effects of conductance changes that could result, for instance, in shunting inhibition were not examined.

In conclusion, we present a comparative study showing how nRT and TC neurons from the adult murine thalamus respond to synthetic EPSCs and IPSCs of different shapes. Our study provides a quantitative map to relate changes in shape parameters of synthetic synaptic currents to neuronal firing in the thalamus, with important implications for interpreting synaptic current changes in normal and pathological conditions.

## Figures and Tables

**Table 1.** Intrinsic properties of nRT and TC neurons

	Membrane Potential Vm, mV	Input Resistance Rm, MΩ*#	Membrane Capacitance Cm, pF*	Tau, ms*	Action Potential Threshold, mV^†^	Action Potential Amplitude, mV^†^	Action Potential Width, ms^†^	Action Potential Half-Width, ms^†^	Tonic Firing Rate, Hz^†^
nRT	−66.07 ± 0.93 (51)	912.03 ± 155.69 (50)	54.70 ± 2.37 (52)	1.12 ± 0.05 (52)	−48.06 ± 0.48 (42)	42.80 ± 1.00 (42)	1.57 ± 0.06 (42)	0.76 ± 0.03 (42)	35.81 ± 2.11 (42)
TC	−69.08 ± 0.69 (54)	362.54 ± 41.20 (51)	99.94 ± 3.95 (51)	2.07 ± 0.09 (51)	−47.87 ± 0.71 (47)	55.32 ± 1.26 (47)	3.04 ± 0.09 (47)	1.51 ± 0.09 (47)	22.89 ± 1.54 (47)
P-value§ (Mann–Whitney test)	**1.75 × 10** ^−**3**^	**<1.00 × 10** ^−**4**^	**<1.00 × 10** ^−**4**^	**<1.00 × 10** ^−**4**^	0.75	**<1.00 × 10** ^−**4**^	**<1.00 × 10** ^−**4**^	**<1.00 × 10** ^−**4**^	**<1.00 × 10** ^−**4**^
VB	−69.71 ± 0.97 (18)	382.35 ± 83.85 (17)	100.73 ± 6.16 (17)	2.09 ± 0.19 (17)	−47.07 ± 1.27 (17)	54.73 ± 2.10 (17)	2.71 ± 0.11 (17)	1.31 ± 0.05 (17)	20.88 ± 2.66 (17)
VL	−69.50 ± 1.12 (25)	360.85 ± 59.76 (23)	102.17 ± 5.44 (23)	2.15 ± 0.10 (23)	−48.51 ± 0.97 (24)	55.58 ± 1.76 (24)	3.34 ± 0.10 (24)	1.69 ± 0.07 (24)	23.75 ± 2.30 (24)
P-value§ (Mann–Whitney test)	0.94	0.43	0.63	0.52	0.47	0.63	**4.67 × 10** ^−**4**^	**<1.00 × 10** ^−**4**^	0.55

Sample size *n* (cells) are included in brackets.

* Measured in response to a 100 ms +5 mV test pulse.

# Atypically high membrane resistance due to the presence of synaptic blockers (i.e., DNQX, APV, picrotoxin) in the bath.

† Action potentials were evoked with 2 s 150 pA injections. Action potential measurements were taken 1 s after the start of injection. Action potential threshold was defined as the Vm when dV/dt measurements first exceeded 15 V/s.

§ The boldface *P* values are significant when considering the Bonferroni-corrected thresholds for multiple comparisons, which is 0.05/9=5.56×10^−3^.

**Table 2.** Spearman correlation coefficients of time to LTS and intrinsic properties of thalamic neurons

	Membrane Potential Vm, mV	Membrane Resistance Rm, MΩ*	Membrane Capacitance Cm, pF*	Tau, ms*	Action Potential Threshold, mV^†^	Action Potential Amplitude, mV^†^	Action Potential Width, ms^†^	Action Potential Half-Width, ms^†^	Tonic Firing Rate, Hz^†^
nRT	−8.96 × 10^−2^ (17)	0.17 (17)	0.35 (17)	0.50 (17)	0.82 (7)	0.54 (7)	0.25 (7)	−0.32 (7)	0.32 (7)
*P*-value§	0.73	0.54	0.17	4.30 × 10^−2^	3.41 × 10^−2^	0.24	0.60	0.48	0.48
TC	−0.18 (17)	5.39 × 10^−2^ (17)	0.27 (17)	0.28 (17)	0.31 (12)	0.36 (12)	0.14 (12)	0.27 (12)	−0.38 (12)
*P*-value§	0.48	0.84	0.30	0.28	0.33	0.25	0.67	0.40	0.25

Sample size *n* (cells) are included in brackets.

* Measured in response to a 100 ms +5 mV test pulse.

† Action potentials were evoked with 2 s 150 pA injections. Action potential measurements were taken 1 s after the start of injection. Action potential threshold was defined as the Vm when dV/dt measurements first exceeded 15 V/s.

§ *P* values are bolded if they are significant when considering the Bonferroni-corrected thresholds for multiple comparisons, which is 0.05/9=5.56×10^−3^. No significant correlations were detected.

## Data Availability

All data and code for the study are available on Zenodo (https://doi.org/10.5281/zenodo.13942488).
